# Disaster Mental Health Risk Reduction: Appraising Disaster Mental Health Research as If Risk Mattered

**DOI:** 10.3390/ijerph20115923

**Published:** 2023-05-23

**Authors:** Lennart Reifels, Michel L. A. Dückers

**Affiliations:** 1 Centre for Mental Health, Melbourne School of Population and Global Health, The University of Melbourne, Parkville, VIC 3010, Australia; 2 Nivel—Netherlands Institute for Health Services Research, 3513 CR Utrecht, The Netherlands; m.duckers@nivel.nl; 3 ARQ Centre of Expertise for the Impact of Disasters and Crises, 1112 XE Diemen, The Netherlands; 4 Faculty of Behavioral and Social Sciences, University of Groningen, 9712 CP Groningen, The Netherlands

## 1. Introduction

The globally increasing frequency, intensity, and complexity of extreme climatic events and disasters poses significant challenges for the future health and wellbeing of affected populations around the world [1,2]. Mental health problems in the general population, which still too often go un-attended or untreated in many countries [3], are known to be further elevated among those exposed to extreme climatic events and disasters [4,5].

In view of the onslaught and rapid succession of such events, affected communities and existing supports are increasingly stretched in their ability and capacity to cope with such events, thus posing urgent questions about how people and support systems can best be enabled to adequately deal with and operate in this changing and challenging reality [6].

Current approaches to addressing the mental health consequences of extreme climatic events and disasters typically involve so-called Mental Health and Psychosocial Support (MHPSS) systems which are informed by guidelines and commonly mobilized during disaster response and recovery phases. These MHPSS systems can involve varied local and external actors who provide supports, ranging from basic emergency support to meet immediate practical and psychosocial needs to advanced psychological treatment, within supportive conditions that globally differ dramatically across geographies, and which can be severely affected by the disaster as well.

The anticipated increase in mental health and psychosocial problems arising from more frequent and cumulative exposure to extreme events and its interplay with demographic, social, economic, and political context-based vulnerabilities, poses several key questions regarding the adequacy of our current MHPSS approaches. These questions foremost concern our understanding of the changing nature and prevalence of mental health impacts that are likely to arise in future, the key strategies needed to more effectively prevent or address these impacts, the associated increases in MHPSS capacity requirements, as well as the traditionally reactive role of MHPSS which is usually limited to addressing mental health impacts after disasters have occurred.

Disaster risk reduction (DRR), as espoused in the Sendai Framework for Disaster Risk Reduction 2015–2030, is a relatively new paradigm which has profoundly shaped the way that contemporary societies deal with disasters through disaster-related policy and practice [7]. DRR moves beyond the traditional approach to managing disaster events and consequences, towards proactive concerted approaches that seek to better understand, reduce, and manage disaster risks. As such, DRR adopts an all-hazard approach, and goes beyond the prevailing event orientation. DRR has found its expression in the health domain within Health Emergency and Disaster Risk Management (Health EDRM) [8].

Yet, until recently, the intersection of DRR and MHPSS has received relatively little systematic attention [9]. Despite a growing recognition of the importance of mental health in global disaster policy (such as the UNDRR Sendai and WHO Health EDRM Frameworks), the intersections of our current approaches to disaster risk reduction and efforts to address mental health and psychosocial aspects in disaster and emergency contexts are still relatively poorly understood. As such, it is pivotal that we deepen our understanding of this intersection and develop the practical and scientific knowledge required to advance this field in the future.

## 2. Special Issue on Disaster Mental Health Risk Reduction

This Special Issue, therefore, provides a unique opportunity to further examine these insufficiently explored themes and intersections of MHPSS and DRR across the various stages of the disaster life cycle and across geographies. The contributions in this Special Issue are brought together through the integrative perspective of Disaster Mental Health Risk Reduction, with the aim to better understand, manage, and reduce future mental health and psychosocial risks associated with disasters and emergencies.

### 2.1. Understanding Disaster Risk

Relatively broad concepts with intuitive appeal, such as “risk” or “disaster risk”, are inevitably open to subjective interpretation, which can give rise to manifold and, at times, conflicting understandings (thus highlighting what could be termed the “arbitrary nature of risk”). In view of this circumstance, it is therefore important to define such key concepts as clearly as possible and in terms that are shared and understood by many if these are to guide our concerted global and everyday efforts to better understand and reduce disaster risk. The UNDRR definition of disaster risk (which comprises the four key elements of hazard, exposure, vulnerability, and capacity) underpins current global policy frameworks (such as the Sendai Framework for Disaster Risk Reduction, the WHO Health EDRM Framework, and the IASC Technical Note on Linking DRR and MHPSS) and provides the most widely accepted definition of this key concept [10]. As such, the following UNDRR definitions were also adopted in this editorial, where they serve as a conceptual basis for examining contributions to the Special Issue.

### 2.2. Definitions

- Disaster risk: The potential loss of life, injury, or destroyed or damaged assets which could occur to a system, society, or a community in a specific period of time, determined probabilistically as a function of hazard, exposure, vulnerability, and capacity. Source: https://www.undrr.org/terminology/disaster-risk (accessed on 28 February 2023).
- Hazard: A process, phenomenon or human activity that may cause loss of life, injury or other health impacts, property damage, social and economic disruption, or environmental degradation. Source: https://www.undrr.org/terminology/hazard (accessed on 28 February 2023).
- Exposure: The situation of people, infrastructure, housing, production capacities, and other tangible human assets located in hazard-prone areas. Annotation: Measures of exposure can include the number of people or types of assets in an area. These can be combined with the specific vulnerability and capacity of the exposed elements to any particular hazard to estimate the quantitative risks associated with that hazard in the area of interest. Source: https://www.undrr.org/terminology/exposure (accessed on 28 February 2023).
- Vulnerability: The conditions determined by physical, social, economic and environmental factors, or processes which increase the susceptibility of an individual, a community, assets or systems to the impacts of hazards. Annotation: For positive factors which increase the ability of people to cope with hazards, see also the definitions of “Capacity” and “Coping capacity”. Source: https://www.undrr.org/terminology/vulnerability (accessed on 28 February 2023).
- Capacity: The combination of all the strengths, attributes, and resources available within an organization, community, or society to manage and reduce disaster risks and strengthen resilience. Annotation: Capacity may include infrastructure, institutions, human knowledge and skills, and collective attributes, such as social relationships, leadership, and management. Source: https://www.undrr.org/terminology/capacity (accessed on 28 February 2023).

**Figure 1:**
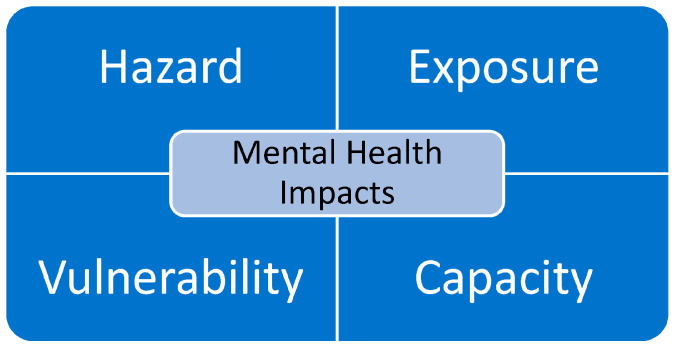
Key elements of disaster mental health risk.

Figure 1 illustrates the four key elements of disaster risk underpinning the UNDRR definition alongside associated “mental health impacts”: a particular particle of “disaster impact”; the total effect, including negative effects (e.g., economic losses); and positive effects (e.g., economic gains) of a hazardous event or a disaster. The term includes economic, human, and environmental impacts, and may include death, injuries, disease, and other negative effects on human physical, mental, and social wellbeing. Source: https://www.undrr.org/terminology/disaster (accessed on 28 February 2023).

Together the key elements form the basic conceptual framework through which the contributions in this Special Issue are being examined. While each of the key elements of this conceptual framework can be addressed at different levels, it is the relative simplicity of this framework and its congruence with existing UNDRR definitions, which facilitate its wider application to understanding disaster risk reduction efforts across varied domains of human endeavor, including those directed at research on mental health and disasters.

### 2.3. Overview of Special Issue Contributions

The 18 articles which make up the body of this Special Issue comprise contributions from Asia, Africa, Europe, North America, and Oceania. They include seminal reviews and empirical studies which address the key elements of disaster mental health risk at individual, community, organization, sector, national/societal, regional, and global levels. The contributions examine mental health risks in the context of disasters marked by specific natural (floods, hurricanes, and earthquakes) and technological hazards (nuclear accidents), as well as in multi-hazard contexts, and global health emergencies (pandemic). Most of the Special Issue contributions were made by researchers with expertise in disaster mental health research or MHPSS, rather than by DRR experts. Table 1 provides an overview of the contributions, which classifies the articles in terms of disaster context, study type, primary disaster risk focus, entity levels addressed, risk reduction strategies, as well as in their broader alignment with the four Sendai priorities for action and 10 Health EDRM functions.

### 2.4. Summary of Key Themes

Key themes resulting from the classification of the articles are briefly summarized below and then discussed in terms of broader research trends and implications.

#### 2.4.1. Risk Elements Addressed

Three key elements of disaster mental health risk, exposure, vulnerability, and capacity were covered extensively in the Special Issue, while one half of the articles also had a primary focus on examining mental health impacts. Disaster mental health impact assessments typically considered exposure and vulnerability variables, which makes sense as disaster mental health risks can only be understood in the context of established disaster health determinants. Primary exposure was frequently established at individual or community levels, yet the degree and type of exposure was not always elaborated to the same detail as suggested in existing reviews [5,11,12,13]. Some authors further disentangled the effect of secondary exposure on mental health, which can be equally substantive, especially when ongoing [14]. Vulnerability was largely considered at the individual level (in terms of sociodemographic and other risk and protective factors), and to a lesser extent at community (socioeconomic status) and national levels (socioeconomic and development indices). Studies addressed capacity at varying levels, through training of individual organizational and sector staff, by substantiating the merit of existing guidelines with wide-ranging applications, and by considering individual level (social support) and community level variables (social capital, collective efficacy) in mental health impact assessments. Physical hazards, perhaps unsurprisingly, rarely formed the explicit focus or object of study in disaster mental health research, where they were mainly operationalized via exposure variables or featured as the study context.

Mental health impact assessment can be regarded as the traditional “strong suit” of disaster mental health research. Accordingly, the nature of mental health impacts examined varied widely, including mental health outcomes, such as depression, anxiety, PTSD, psychological distress, and negative emotional expressions. Predominantly, mental health impacts were assessed at the individual and community levels, while some studies also considered the multi-level structure of the data to verify whether mental health risks were linked to country characteristics or specific geographical and vulnerability contexts [15,16].

Research approaches underpinning such assessments were primarily cross-sectional and correlational, and often depending on self-reporting. Therefore, whilst informative, caution remains necessary, since the lack of pre-event baseline data does not permit ascertaining the relative disaster impact, and samples are difficult to compare because of heterogeneity in explicit risk and protective factors that are accounted or controlled for, and those factors which remain implicit.

#### 2.4.2. Risk Reduction Strategies

Key strategies adopted in studies to reduce disaster mental health risks primarily focused on fostering DRR-MHPSS integration in policies and guidelines, improving our understanding of the nature of disaster mental health risks (e.g., in terms of underpinning exposure and vulnerability drivers), surveillance and assessment of resulting disaster mental health impacts, and capacity building via training and education initiatives. While intervention studies and disaster mental health services research did not directly feature in Special Issue contributions, both areas of research clearly warrant further attention as important avenues for disaster mental health risk reduction [17,18].

#### 2.4.3. Sendai and Health EDRM Alignment

In terms of their alignment with Sendai priorities for action, most primary studies were concerned with understanding disaster risk (Sendai Priority Action 1), while four training studies also focused on enhancing disaster preparedness (Sendai Priority Action 4). The three reviews of MHPSS guidelines and DRR-MHPSS integration domains and practices sought to strengthen disaster risk governance (Sendai Priority Action 2) and enhance disaster preparedness (Sendai Priority Action 4). While MHPSS guidelines also encourage investment, none of the studies in the Special Issue explicitly focused on investing in disaster risk reduction (Sendai Priority Action 3).

Of the 10 Health EDRM functions, the three reviews primarily addressed policies, strategies, and legislation (function 1); planning and coordination (function 2); and monitoring and evaluation (function 10); while also contributing to information and knowledge management (function 5) via development of good practice guidelines. Most primary studies contributed directly to information and knowledge management (function 5) via empirical research for Health EDRM, and, to a lesser extent, to human resources (function 3) via curriculum development and training delivery, and monitoring and evaluation (function 10) of assessment tools and training courses. Other Health EDRM functions (including financial resources; risk communication; health infrastructure and logistics; health and related services; and community capacities for Health EDRM) received relatively little explicit attention in Special Issue contributions, thus highlighting promising areas for future primary research, systematic review, or further integration.

**Table 1:** Overview and classification of Special Issue contributions.

Article	Article Synopsis	Disaster Context	Study Type	Primary Disaster Risk Focus	Entity Level Addressed	Risk Reduction Strategy Focus	Sendai Priority for Action (1–4)	Health EDRM Function (1–10)
1. Gray et al. (2020) [19]	Mapping and review of DRR-MHPSS integration domains and practices	Multi-hazard	Review	Hazard Exposure Vulnerability **Capacity**	Individual **Community** **Organization** **Sector** **National**/**societal** **Regional** Global	Policy/Guidelines (DRR-MHPSS integration)	2, 4	1, 2, 10
2. Te Brake et al. (2022) [20]	Assessment of the methodological quality of MHPSS guidelines	Multi-hazard	Review	Hazard Exposure Vulnerability **Capacity**	Individual Community **Organization** **Sector** **National**/**societal** **Regional** **Global**	Policy/Guidelines (DRR-MHPSS integration)	2, 4	1, 2, 5, 10
3. Dückers et al. (2022) [21]	Assessment of the content of MHPSS guidelines	Multi-hazard	Review	Hazard Exposure Vulnerability **Capacity**	Individual Community **Organization** **Sector** **National**/**societal** **Regional** **Global**	Policy/Guidelines (DRR-MHPSS integration)	2, 4	1, 2, 5, 10
4. McKenzie et al. (2022) [14]	Cross-sectional survey of mental health impacts and secondary stressors in flood-affected communities	Post-disaster (floods)	Empirical study	Hazard **Exposure** **Vulnerability** Capacity **Disaster MH impacts**	**Individual** **Community** Organization Sector National/societal Regional Global	Understanding DMH risks DMH impact assessment	1	5
5. Carl et al. (2022) [22]	Psychometric study to determine Post-Hurricane Distress Scale cut-off scores	Post-disaster (hurricanes)	Empirical study	Hazard **Exposure** **Vulnerability** Capacity **Disaster MH impacts**	**Individual** **Community** Organization Sector National/societal Regional Global	Understanding DMH risks DMH impact assessment	1	5, 10
6. Garske et al. (2021) [23]	Spatial epidemiological study of negative emotional expressions on Twitter and associations with area demographics and hurricane damage	Pre-peri-post disaster (hurricane)	Empirical study	Hazard **Exposure** **Vulnerability** Capacity **Disaster MH impacts**	**Individual** **Community** Organization Sector National/societal Regional Global	Understanding DMH risks DMH impact assessment	1	5
7. Généreux et al. (2020) [24]	Multi-country, cross-sectional survey of mental health impacts and risk and protective factors during the COVID-19 pandemic	Peri-disaster (pandemic)	Empirical study	Hazard **Exposure** **Vulnerability** Capacity **Disaster MH impacts**	**Individual** Community Organization Sector **National**/**societal** **Regional** Global	Understanding DMH risks DMH impact assessment	1	5
8. Généreux et al. (2021) [15]	Multi-country, repeated cross-sectional survey of mental health impacts and risk and protective factors during the COVID-19 pandemic	Peri-disaster (pandemic)	Empirical study	Hazard **Exposure** **Vulnerability** Capacity **Disaster MH impacts**	**Individual** Community Organization Sector **National**/**societal** **Regional** Global	Understanding DMH risks DMH impact assessment	1	5
9. Harigane et al. (2021) [25]	Cross-sectional survey examining levels of and factors hindering social participation among nuclear accident evacuees	Post-disaster (nuclear accident)	Empirical study	Hazard **Exposure** **Vulnerability** Capacity	**Individual** **Community** Organization Sector National/societal Regional Global	Understanding DMH risks	1	5
10. Pandit et al. (2021) [26]	Cross-sectional survey examining the cumulative effects of reciprocal social support on depression in earthquake affected district	Post-disaster (earthquake)	Empirical study	Hazard **Exposure** **Vulnerability** **Capacity** **Disaster MH impacts**	**Individual** **Community** Organization Sector National/societal Regional Global	Understanding DMH risks DMH impact assessment	1	5
11. Wind et al. (2021) [27]	Cross-sectional survey examining multi-level social mechanisms of depression following floods	Post-disaster (floods)	Empirical study	Hazard **Exposure** **Vulnerability** **Capacity** **Disaster MH impacts**	**Individual** **Community** Organization Sector National/societal Regional Global	Understanding DMH risks DMH impact assessment	1	5
12. Evans et al. (2021) [28]	Development and pre/post evaluation of disaster mental health training in terms of knowledge, skills, and attitudes	Multi-hazard	Empirical study	Hazard Exposure Vulnerability **Capacity**	**Individual** Community Organization **Sector** National/societal Regional Global	Training/Education (DRR-MHPSS integration)	4	3, 10
13. Matthews et al. (2020) [29]	Cross-sectional survey examining associations between flood impact, social capital and psychological distress in diverse rural community	Post-disaster (floods)	Empirical study	Hazard **Exposure** **Vulnerability** **Capacity** **Disaster MH impacts**	**Individual** **Community** Organization Sector National/societal Regional Global	Understanding DMH risks DMH impact assessment	1	5
14. Hidaka et al. (2020) [30]	Pre/post training evaluation examining association between anxiety over radiation exposure and occupational health management knowledge	Post-disaster (nuclear accident)	Empirical study	Hazard **Exposure** Vulnerability **Capacity**	**Individual** Community **Organization** Sector National/societal Regional Global	Understanding DMH risks Training/Education	1, 4	3, 10
15. Orui et al. (2020) [31]	Development of training on radiation health anxiety and mental health issues after nuclear disaster; pre/post/follow up evaluation of counselling confidence	Post-disaster (nuclear accident)	Empirical study	Hazard **Exposure** Vulnerability **Capacity**	**Individual** Community **Organization** Sector National/societal Regional Global	Training/Education	4	3, 10
16. Knipscheer et al. (2020) [16]	National cross-sectional survey examining prevalence of potentially traumatic and other life events, PTSD and risk and protective factors	Multi-hazard	Empirical study	Hazard **Exposure** **Vulnerability** Capacity **Disaster MH impacts**	**Individual** Community Organization Sector **National**/**societal** Regional Global	Understanding DMH risks DMH impact assessment	1	5
17. Sijbrandij et al. (2020) [32]	Cluster-RCT examining PFA training effectiveness on health unit staff knowledge and understanding of psychosocial support principles and skills following adversity	Multi-hazard	Empirical study	Hazard Exposure Vulnerability **Capacity**	**Individual** Community **Organization** Sector National/societal Regional Global	Training/Education	4	3, 10
18. Comtesse et al. (2021) [33]	Explores ecological grief as a functional and/or maladaptive response to environmental change, outlining future directions for disaster mental health and bereavement research	Multi-hazard	Perspective	**Hazard** Exposure **Vulnerability** Capacity	**Individual** Community Organization Sector National/societal Regional Global	Understanding DMH risks	1	5

Note. The above classification of Special Issue articles reflects the analytical perspective adopted by the guest editors and not necessarily the views of contributing authors. The Sendai Framework for Disaster Risk Reduction Priorities for Action include: 1. Understanding disaster risk, 2. Strengthening disaster risk governance, 3. Investing in disaster risk reduction, and 4. Enhancing disaster preparedness [7]. Health EDRM key functions include: 1. Policies, strategies, and legislation; 2. Planning and coordination; 3. Human resources; 4. Financial resources; 5. Information and knowledge management; 6. Risk communications; 7. Health infrastructure and logistics; 8. Health and related services; 9. Community capacities for Health EDRM; and 10. Monitoring and evaluation (see WHO 2019, Annex 2).

## 3. Reflection on the Current State of Knowledge in the Field

This Special Issue offers a snapshot of how academics from this multi-faceted field of research—under the growing realization that the probable mental health impact of a world facing multiple global developments and tensions, on top of existing local sources of exposure and adversity—are assembling distinct pieces of a DMHRR jigsaw puzzle that needs to fall into place. Risk reduction strategies with an emphasis on mental health can only be meaningful when they address those elements of hazards, exposure, vulnerabilities, and capacity that have been confirmed to be linked to disaster mental health impacts.

An honest assessment of the current state of knowledge can only lead to the conclusion that, firstly, important progress is made on indispensable pieces of the puzzle and that some pieces are more detailed than others and richly prevalent. This applies to understanding disaster mental health risks. Despite variations in sampling methods, measurement instruments, and statistical analysis techniques, contemporary studies in this Special Issue fit well within already existing knowledge.

Secondly, other puzzle pieces are starting to materialize, but still lack detail. This applies to MHPSS guidelines, bringing together evidence-informed knowledge on how to address these disaster mental health risks, especially when it comes to examples of how they can be implemented effectively in different population contexts. In this respect, this Special Issue contains promising examples of how training can contribute to improved knowledge and skills of participants.

Nevertheless, it also spotlights crucial pieces of the puzzle that are still missing. Namely, the effectiveness of policy documents, guidelines, training programs, and other tools and good practices when they are implemented; to what extent do they—despite their inherent logic, appeal and promise—lead to a reduction in disaster mental health risks in practice? This is linked distinctively to the key theme “capacity”. The contributions in this Special Issue approach capacity via document analysis (the review studies), training evaluations (focusing on participants), and disaster mental health risk assessments (epidemiological studies). Although their importance is not to be disputed, a crucial weak spot is that the first contains little information on how it might work in different vulnerability contexts, the second runs short in providing evidence that the acquired knowledge and skills by participants really result in a better capacity to respond to disasters and ultimately positive outcomes, and the third lacks information on how exogenous variables, such as social capital or support, can be influenced. Other noteworthy missing pieces include the fact that, clearly, not all the Sendai Priority Actions and Health EDRM functions receive an equal amount of scholarly attention. When DMHRR research can focus on issues such as these, it would entail a crucial step in strengthening the knowledge base as well as the practical potential for risk reduction.

Thus, undeniably, important progress is made in understanding the mental health impact of disaster exposure, additional losses, and the interplay with risk factors that increase vulnerabilities at different levels across countries and regions, linked to the capacity to adapt or recover. A willing observant might conclude that a complementary common ground of evidence-based knowledge to shape and direct this capacity is embedded in available guidelines and policy documents, partially translated into practical training programs with promising first results. Yet, how to close the circle and actually reduce such risk tends to be an untested hypothesis. As such, when we envisage the current state of the art as a box with pieces of the DMHRR puzzle, we have to work with a set of pieces in different stages of completeness; the first half depending heavily on mental health expertise and the second part requiring assistance from a broad range of disciplines, specialized in implementation science, risk reduction, and preparedness studies to fill the gaps of the continuing puzzle.

## 4. Advantages and Limitations of the Risk Perspective

Disaster risk is central to DRR, yet risk (in whatever conceptualization) is not a primary research concept espoused or the explicit focus of study in most Special Issue contributions. This observation may be reflective of wider research trends in this field and, arguably, in the broader domain of disaster-related health research. Therefore, just as capacity constitutes the often-neglected element in research on disaster risk, disaster risk itself can be identified as the missing key concept in current disaster mental health research (from a DRR perspective). This is notwithstanding colloquial references to risk or the prevailing focus on risk and protective factors. The absence of an explicit focus on disaster risk, however, does not mean that valuable study contributions are not compatible with DRR or that these could not be integrated within that broader paradigm, as we have sought to demonstrate.

Disaster mental health risk is an integral facet of disaster risk more generally, and entirely congruent with the broader conceptual vocabulary underpinning the UNDRR definitions. As such, disaster mental health risk comprises the same key elements of disaster risk, which are applied in relation to the likelihood of a specific subset of disaster-related health impacts, consequences, or losses.

Based on these basic disaster risk elements or building blocks, it is further possible to consider different types of disaster risks. Some authors differentiate the risk or likelihood of the disaster itself occurring from the risk or likelihood of disaster-related health impacts or consequences, noting that both are characterized by a mismatch of disaster-induced needs (caused by hazard exposure) which exceed available resources of the population (capacity) [34]. Others highlight that beyond primary risks of newly emerging or exacerbated mental health issues among disaster-affected populations, disaster mental health risks also exist in relation to adverse impacts on mental health support systems, which can equally shape mental health outcomes [35,36]. However, crucially, while the building blocks can be combined into formulae and addressed at different entity levels, it is, in essence, the same basic elements that make up our disaster risk vocabulary, irrespective of the specific domain under study.

“Disaster risk” can also appear a somewhat abstract notion (if understood as the probability of adverse impacts resulting from the interplay of its four key elements). Clarity, simplicity, and a shared understanding of such concepts are, therefore, key to coherent knowledge integration in our field. As we argue in this editorial, placing the concept of disaster risk at the heart of DMHRR can serve to locate and integrate specific research contributions, identify current gaps and emphases, and inform future research priorities.

## 5. Challenges, Gaps, and Ways Forward

The Sendai Midterm Review explores global efforts to integrate disaster risk reduction into decision-making, investment, and behaviour that spans sectors, disciplines, geographies, and scales, so as to prompt re-examination and redress of the relationship with risk [37]. The review findings indicate that considerable strides have been made in implementing the Sendai Framework and particularly in relation to our understanding of disaster risks. Yet, global progress remains variable and is significantly hampered by unattended disaster risks which increasingly manifest in adverse impacts that affect a growing number of people, as well as by the often complex and interconnected nature of disaster risks and underpinning risk drivers. Thus, while understanding disaster risks will remain important and a lot of progress has been made in this respect, it is also clear that momentum needs to shift towards the design and implementation of effective strategies to better manage and reduce disaster risks.

Against this backdrop, and in view of associated mental health impacts, it is vital that we foster the integration of DRR and MHPSS and expand the existing body of MHPSS knowledge regarding the evolving nature and scale of disaster mental health risks along with more effective strategies to address or reduce these [9]. The proactive approach underpinning DRR can herein help to widen the traditionally narrow window and spectrum of post-disaster mental health intervention to incorporate a broader array of DMHRR strategies that are applied at varying stages and levels. To this end, Sendai priorities for action and Health EDRM functions can provide valuable guidance to orientate the development of future DMHRR strategies, which, in turn, will serve to further strengthen, expand, and harness the contribution of MHPSS for disaster risk reduction.
